# Machine learning, transcriptome, and genotyping chip analyses provide insights into SNP markers identifying flower color in *Platycodon grandiflorus*

**DOI:** 10.1038/s41598-021-87281-0

**Published:** 2021-04-13

**Authors:** Go-Eun Yu, Younhee Shin, Sathiyamoorthy Subramaniyam, Sang-Ho Kang, Si-Myung Lee, Chuloh Cho, Seung-Sik Lee, Chang-Kug Kim

**Affiliations:** 1 Genomics Division, National Institute of Agricultural Sciences, Jeonju, 54874 Korea; 2 Research and Development Center, Insilicogen Inc., Yongin-si 16954, Gyeonggi-do, Republic of Korea; 3 Crop Foundation Research Division, National Institute of Crop Science, RDA, Wanju, 55365 Korea; 4 Advanced Radiation Technology Institute, Korea Atomic Energy Research Institute, 29 Geumgu-gil, Jeongeup, 56212 Korea; 5 Department of Radiation Science and Technology, University of Science and Technology, Daejeon, 34113 Korea

**Keywords:** Computational biology and bioinformatics, Plant sciences

## Abstract

Bellflower is an edible ornamental gardening plant in Asia. For predicting the flower color in bellflower plants, a transcriptome-wide approach based on machine learning, transcriptome, and genotyping chip analyses was used to identify SNP markers. Six machine learning methods were deployed to explore the classification potential of the selected SNPs as features in two datasets, namely training (60 RNA-Seq samples) and validation (480 Fluidigm chip samples). SNP selection was performed in sequential order. Firstly, 96 SNPs were selected from the transcriptome-wide SNPs using the principal compound analysis (PCA). Then, 9 among 96 SNPs were later identified using the Random forest based feature selection method from the Fluidigm chip dataset. Among six machines, the random forest (RF) model produced higher classification performance than the other models. The 9 SNP marker candidates selected for classifying the flower color classification were verified using the genomic DNA PCR with Sanger sequencing. Our results suggest that this methodology could be used for future selection of breeding traits even though the plant accessions are highly heterogeneous.

## Introduction

The bellflower (*Platycodon grandiflorus*) is a popular plant used as food, medicine, and ornamental plant in Asia^1^. *P. grandiflorus* is a monotypic species of the bellflower family (Campanulaceae). Due to its therapeutic effects, its root has been used in traditional medicine as a popular food additive for over 2000 years^2^. *P. grandiflorus* consists of 12 cultivars, which has received high commercial ratings in the ornamental flower market for their habitat and floral displays^3^. Its flower is characterized by an attractive colorful bud, a long flowering time, and an extended vase life^4,5^.

Genome-wide molecular markers-based genomic selections enhance plant breeding to produce the desired traits^6^. However, traditional molecular breeding techniques, such as molecular assisted selection, have been limited due to multiple gene variants for complex traits^7^. Next-generation sequencing technologies have enabled large-scale genome-wide genotyping for heterogeneous phenotypes, which helped in precise genome selection associated with specific phenotypes. Further, the reduced representation of genome-wide genotyping is transcriptome-wide association studies, which leverage the project cost for molecular breeding studies in the model and non-model plants^8–10^. These massive genotyping efforts have recently been subject to machine learning (ML) methods to predict SNP associations with specific traits. Population studies have demonstrated how ML based modeling can be used effectively to predict phenotype from genotype^11^. ML facilitates pattern recognition of large biological datasets since ML algorithms are used widely in various biological fields, such as molecular marker identification, coding region recognition, pathway gene recognition, protein–protein interaction determination, and metabolic network detection^12^. For instance, ML models have been constructed for genomic selection in wheat^13^, root genotype classification^14^, nut-size prediction in *Castanea crenata*^10^, and polyploidy associated SNPs identification in plants^15^.

Several studies have been conducted in *P. grandifloras* to identify molecular markers such as simple sequence repeats^16,17^, microsatellites^18^, and cleaved amplified polymorphic sequences^19^. However, molecular markers that determine specific flower colors are limited. Hence, we report in this study a newly designed transcriptome-wide approach as an effective method in identifying flower color in bellflower plants, where the selected SNP markers could be used to predict bellflower color during the plant breeding experiments.

## Methods

### Plant materials

*P. grandiflorus* plant seeds germinated at 60-hole pots for 30 days and transferred to 18.3 CM PI-pots for 90 days in green house setup under 25 °C temperature. Once the flower color was conformed, the petals were collected as a samples for experiments. All the plants used in this study were maintained at experimental field located in Jeonju, Korea (N: 35° 49′; E: 127° 09′) National Institute of Agricultural Sciences/RDA living modified organism (LMO) guidance. Accessions for three flower colours (i.e., Astra pink, Janbaek violet, and Jangbaek white) for RNA sequencing (RNA-Seq) were obtained from the RDA Genebank (http://genebank.rda.go.kr/). For three colors (20 in each color), 60 samples were collected, and those sampling details are given in Supplementary Table S2. The 480 genotyping samples for the Fluidigm chip array were collected from the mutant accessions created by the gamma irradiation of three different flower color accessions (Supplementary Table S3). The leaf and flower components from each plant were collected individually, and the samples were frozen in liquid nitrogen and stored at -80℃ following DNA and RNA extractions.

### Gamma irradiation

A Co-60 gamma-irradiator (IR 222, MDS Nordion Inc., Kanata, Canada) was used for gamma irradiation. Dry seeds of *P. grandiflorus* were irradiated with 50, 100, 150, and 200 Gy of gamma radiation at dose rates of 25, 50, 75, and 100 Gy h^–1^ for 2 h using a Co-60 gamma-irradiator at the Advanced Radiation Technology Institute (ARTI), Korea Atomic Energy Research Institute (KAERI).

### *P. grandifloras* reference genome

The reference genome for *P. grandifloras* was obtained from the *P. grandiflorus* genome project^1^ (http://platycodon.theragenetex.com/). The gene model and other functional annotations were also obtained from the respective site.

### RNA sequencing and variant calling

The 60 transcriptome sequence libraries were subjected to a high-throughput Illumina NovaSeq sequencing system for RNA sequencing. RNA was extracted from individual samples using TRIzol reagent (Invitrogen, Thermo Fisher Scientific, Waltham, MA, USA). Total RNA was quantitated using the NanoDrop spectrophotometer (Invitrogen, Thermo Fisher Scientific, Waltham, MA, USA), and quality was assessed using the RNA 6000 Nano assay kit and Bioanalyser2100 (Agilent Technologies, Santa Clara, CA, USA).

Before variant calling, sequence reads were checked for bacterial contamination and adapters using Trimmomatic v0.36^20^, as explained in shin et. al.,^21^ and mapped to the reference genome using bowtie2^22^. To optimize the small insertion and deletion artifacts, the reads were re-mapped to the reference genome using the GATK v3.5 tool^23^ and the variants for individual samples were stored in the variant call format (VCF) files. The command line includes the parameters (T:HaplotypeCaller; emitRefConfidence:GVCF, variant_index_type:LINEAR, varient_index_parameter:128,000; nct:20; drf:DuplicateRead). Using filters such as the normalized quality score ≥ 20 and mapping quality ≥ 40, the high-quality SNPs were obtained. The obtained SNPs were annotated using SnpEff v4.2^24^ and the missing genotypes were imputed by Beagle v4.1^25^ with the linkage disequilibrium (LD) score.

### Population stratification

For population stratification, SNPs were examined via principal component analysis (PCA) using the PLINK v1.9 tool^26^. The commands include options such as assoc, adjust, fisher, model, logistic, hap-assoc, hap-impute. To reduce false positive predictions, stringent filtering cutoffs such as genotyping rate ≥ 90%, mapping quality ≥ 40, minor allele frequency (MAF) > 5%, and Hardly-Weinberg equilibrium (HWE) < 0.001were applied to the scores calculated by the PLINK tool. The sub-populations were estimated based on the number of clusters (i.e., K value), which was obtained using the STRUCTURE v2.3.4 tool^27^. The ad-hoc static *ΔK* (i.e., change rate of log probability between successive K values) was used to determine the uppermost hierarchical level of population structures. Structural analysis was performed following 20 replicated runs using the 100,000 iterations after a burn-in period of 50,000 runs.

### Genome-wide association

**Figure 1:**
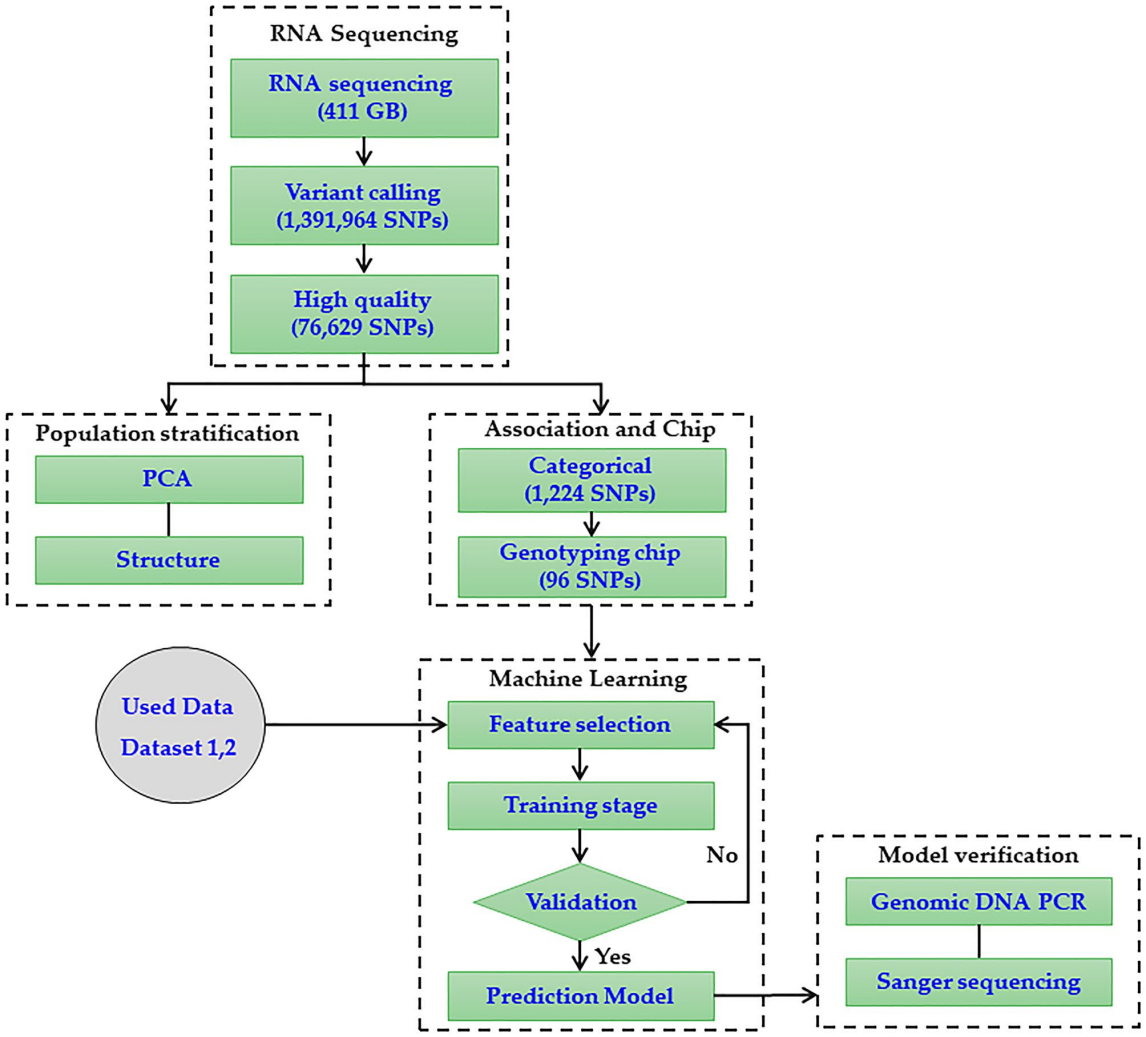
Overview of the SNP analysis pipeline for *P. grandiflorus* flower color classification using machine learning models.

A genome-wide association study was conducted with categorical association (case vs. control). Here, there are three flower color categories tread as case vs control (example: white vs other two as controls). The association between genotype and phenotype for the above model was conducted using PLINK-v.1.9 with the association mode (Fig. 1). The significant SNPs were selected by applying the cut-off, *p* < 0.01.

### Genotyping chip construction

The SNPs for Fluidigm chip manufacturing were selected in two steps from the color-associated SNPs obtained through GWAS. Firstly, 50% of SNPs were systematically selected from mapping feature values such as transcripts per millions (TPM) ≥ 0.3, Read counts ≥ 5), differentially expressed genes/transcripts (log_2_FC) ≥ 2 among the color-specific sets with others, and false discovery rate (FDR) < 0.05, and ± 5 Kb of flanking regions. Another 50% were manually selected by the reported flower color-associated genes. The SNPs were identified from the reported anthocyanin pathway and gene expression profile of the *P. grandiflorus* tissues*.* Finally, a Fluidigm genotyping chip was designed using 96 SNPs (49 systematically selected SNPs and 47 manually selected SNPs). These SNPs belong to the 75 genes present in the reference genome (Supplementary Table S4).

Validation of single-base polymorphism genotyping was conducted using the targeted allele- and locus-specific primers with 96.6 dynamic array-integrated fluidic circuit technology in a Fluidigm bio-mark HD system (Fluidigm, South San Francisco, CA, USA). DNA integrity was first assessed by spectrophotometry at 260/280 nm and quantification of DNA concentration (ng/ul) using a NanoDrop spectrophotometer (Invitrogen, Thermo Fisher Scientific, Waltham, MA, USA). One Fluidigm SNP chip contains allele-specific primer 1, 2, locus-specific primer, and specific target amplification primer. Furthermore, the probe for individual SNPs was prepared as per the manufacturer’s instructions and the bi-allele fluorescence signals were captured using a Fluidigm chip-compatible instrument.

### Prediction and evaluation of ML models

Six supervised ML algorithm models were used to estimate the effectiveness of the selected SNP features. The six models used were support vector machine (SVM), *k*-neural network (*k*-NN), random forest (RF), C5.0 decision tree (C5.0), partial least square (PLS), and gradient boosting (GBM). The training population in each dataset was divided into a training dataset and a validation dataset at a 7:3 ratios for the prediction models. The best model was selected automatically by the caret package^28^. Here we used the caret R package to train (with number = 1000, classProbs = True, savePredictions = True, p = 0.7) and predict (type = "prob") functions with default values other than custom parameters used in brackets. For the feature prioritization we used varImp function only with random forest method. To compare the prediction methods, we determined sensitivity, specificity, and accuracy, using the following equations: Sensitivity = [TP/(TP + FN)]; Specificity = [TN/(TN + FP)]; and Accuracy = [(TP + TN)/ (TP + FP + TN + FN)]; where TP was the number of true positives, TN was the number of true negatives, FP was the number of false positives, and FN was the number of false negatives. The performances of the prediction models were assessed using ROC curves, plotting the sensitivity as a function (1-specificity) for different decision thresholds. Further, to quantitatively compare the ROC curves, we computed the AUC, and significant differences between two ROCs were assessed using a two-tailed Student’s t-test. These evaluation metrics were calculated as explained by Kang e.al^10^. To calculate the ROC and the AUC, we used the plotROC package^29^. The six ML models for flower color prediction were estimated using two datasets. Dataset 1 consisted of training using 60 RNA-Seq data and validation with 480 Fluidigm data. Dataset 2 consisted of training using RNA-Seq + 40% Fluidigm data (n = 252) and validation with 60% Fluidigm data (n = 288). For the training stage, 40% of samples were randomly selected from all Fluidigm samples (Supplementary Table S3).

### Validation of candidate SNPs by genomic DNA PCR and Sanger sequencing

Genomic sequences, including forward and reverse 500-bp flanking regions of the candidate SNPs, were extracted from the reference genome of *P. grandiflorus*^30^. Genomic DNA was extracted from the leaves of *P. grandiflorus* plants with pink, violet, and white flowers using the general cetyltrimethylammonium bromide (CTAB) method. Genomic DNA PCR was performed using the following conditions: 95 °C for 5 min; followed by 35 cycles of 95 °C for 30 s, 55 °C for 30 s, and 72 °C for 1 min; and a final extension at 72 °C for 7 min. Afterward, PCR amplicons were separated by 1.5% agarose gel electrophoresis and visualized by staining with EtBr solution. Representative PCR amplicons for each of the three flower color plants were selected and subjected to ABI Sanger sequencing using an ABI 3730xl System (Macrogen, Seoul, Korea). The nucleotide sequences of PCR amplicons were assembled using SeqMan pro (DNASTAR, Madison, WI, USA) with default parameters.

## Results

### Sequencing and variant calling

A total of 411 Gb of RNA sequence data was produced from 60 tissue samples of leaf and flower components in three accessions from plants with pink, violet, and white flowers (Fig. 1). After trimming for quality, 373.5 Gb (average 6.2 Gb/sample) of sequencing data were finally obtained, and 88.1% of reads were mapped to the *P. grandiflorus* reference genome (Supplementary Fig. S1). These mapping reads covered 29,385 (65.3%) genes in the *P. grandiflorus* reference genome. A total of 1,391,964 SNPs were identified using the GATK variant call procedure, and 76,629 high-quality SNPs were found from the PLINK filtration procedure. The PLINK categorical association procedure identified 1,224 flower color-associated SNPs from high-quality SNPs (p-value < 1e^-10^).

To manufacture the mass genotyping chips, we identified 96 SNPs from the total color- associated SNPs. These 96 SNPs consisted of 49 that were systematically selected and 47 that were manually selected. The 49 systematically selected SNPs were identified from genotyping criteria based on chip design protocols (i.e., TPM ≥ 0.3, Read count ≥ 5, log_2_FC ≥ 2.0, and 5 Kb up/down flanking regions). The selected SNPs were tested for their associated relationship with the flower color using PCA and heat-map analysis. The results were clustered efficiently into three flower color groups with minor allelic frequency (Supplementary Fig. S2). The 47 manually selected SNPs were identified based on the anthocyanin biosynthesis pathway and flower color-associated gene expression profiles generated from the *P. grandiflorus* tissue samples (Supplementary Fig. S3).

### Genetic diversity and population structure

This study’s samples were highly heterogeneous and assessed by population stratification, hetero/homo allele ratio, and genetic diversity analysis. The population structure was estimated using *K* populations based on a maximum likelihood method. Although the optimal number of groups was three (*K* = 3), population stratification demonstrated that each of the three groups included different flower color sub-groups (Supplementary Fig. S4). Genetic diversity was assessed using the 76,629 high-quality SNPs and 1,224 flower color-associated SNPs. PCA showed that two SNP groups could not be clearly classified into the three genotype groups based on flower color, namely white, violet, and pink (Supplementary Fig. S5). The hetero/homo allele ratio was estimated from 1,624,281 variants (i.e., 1,391,964 SNPs, 110,687 insertion polymorphisms, and 121,630 deletion polymorphisms), which were generated using the GATK variant call procedure with mapping quality ≥ 40. The hetero/homo allele ratio variation was most significant in the white group according to all the variant types (i.e., SNP and insertion and deletion polymorphism) (Supplementary Fig. S6).

### Prediction and validation of Fluidigm genotyping chip

The efficiency of manufactured Fluidigm genotyping chips was tested at two dataset stages (i.e., dataset 1 and dataset 2) in two steps, namely training and validation process. For the first dataset stage (i.e., dataset 1), which consists of 96 SNPs, 60 transcriptome data of the three flower color types were used to train six ML models, and 480 chip data were validated using the same models. A total of 540 samples were evaluated using the ML model. The gamma irradiation mutants produced 480 verification samples. These mutant plants with distinct molecular properties make it possible to gain further insight into the relationship between the main regulatory processes^31^. These mutant samples shared a similar genetic background across the transcriptome samples even though they exhibited different phenotypes except for the flower color. These mutants were then utilized to compare the gene expression patterns related to flower color pathways.

The prediction accuracy of the six models was low with an average value of 0.601 (Table 1). The classification potential for flower color was assessed using three factors (i.e., balanced accuracy, sensitivity, and specificity) of the ML model. Varying the features from 3 to 96 SNPs did not efficiently predict the three factors of the six ML models (Supplementary Fig. S7). Hence, we assumed that ML models exhibited a low prediction efficiency because the validation samples had a varying phenotype in the gamma irradiation mutants.

**Table 1:** Predictions accuracy of six ML models on the two datasets.

Stage	Model	First (96 SNPs)	Second (9 SNPs)	Average
Dataset 1	GBM	0.617	0.752	0.684
	SVM	0.479	0.760	0.620
	RF	0.646	0.777	0.711
	KNN	0.640	0.727	0.683
	C5.0	0.604	0.721	0.663
	PLS	0.617	0.752	0.684
Dataset 2	GBM	0.705	0.774	0.740
	SVM	0.840	0.757	0.799
	RF	0.819	0.799	0.809
	KNN	0.778	0.743	0.760
	C5.0	0.816	0.781	0.799
	PLS	0.705	0.774	0.740

To improve the prediction accuracy, the feature importance (among the total SNPs, which subset of SNPs have higher prediction potentials, while subject as features to the machines), the value was predicted using the random forest (RF) model algorithm. The feature importance value indicated the ability to distinguish the three flower color groups, and these values generally appeared in a slightly decreasing pattern (Supplementary Fig. S8). This approach identified 9 SNPs (i.e., greater than 2.5 on the y-axis). The heat-map displaying results from the 9 SNPs shows that flower color can be discriminated and that the different positional alleles exist within the same group (Supplementary Fig. S9).

**Figure 2:**
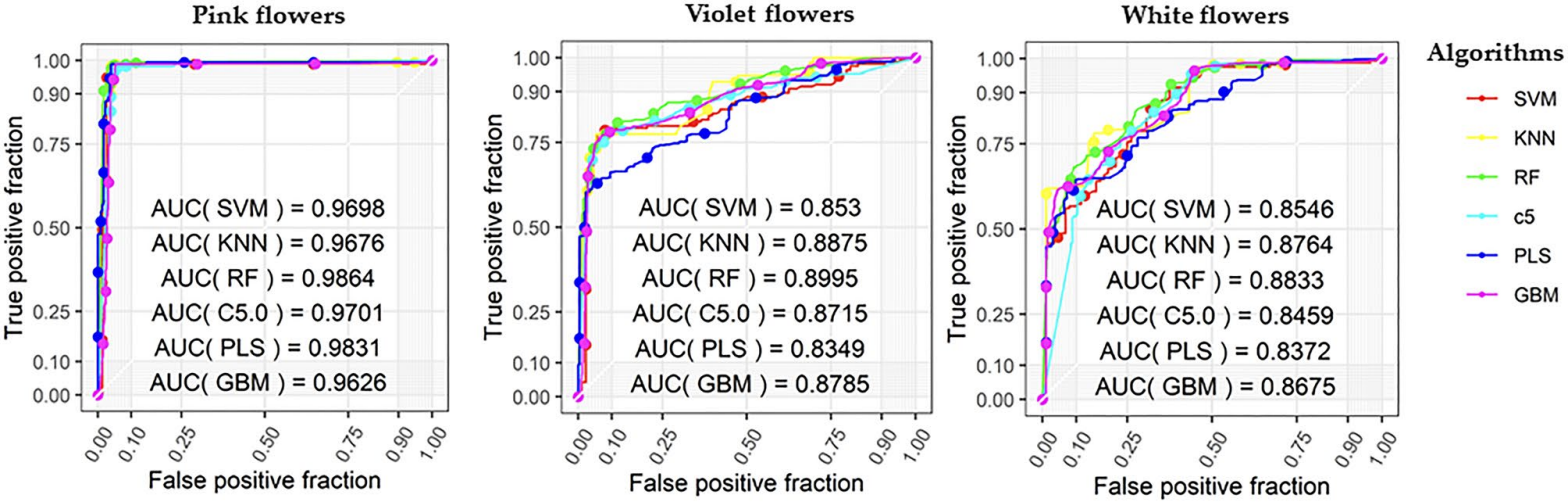
Machine learning model for flower color prediction accuracy in *P. grandiflorus*. Receiver operating characteristic (ROC) curves for 9 SNPs (FDR < 0.05) using six machine learning models. Each values of area under the curve (AUC) shows the average of 10 cross validations for pink, violet, and white flowers. The six models used are support vector machine (SVM), *k*-neural network (*k*-NN), random forest (RF), C5.0 decision tree (C5.0), partial least square (PLS), and gradient boosting (GBM).

For the second dataset stage (i.e., dataset 2) using the 9 SNPs, 252 training samples were tested, and 288 genotyping chip samples were validated using the same models. The prediction accuracy had an average value of 0.771, which is an improvement over that of 96 SNPs (Table 1). The receiver operating characteristic (ROC) curves for the 9 SNPs (FDR < 0.05) shows that the RF model had the highest accuracy and that the pink flower type was predicted with the highest efficiency (Fig. 2).

**Figure 3:**
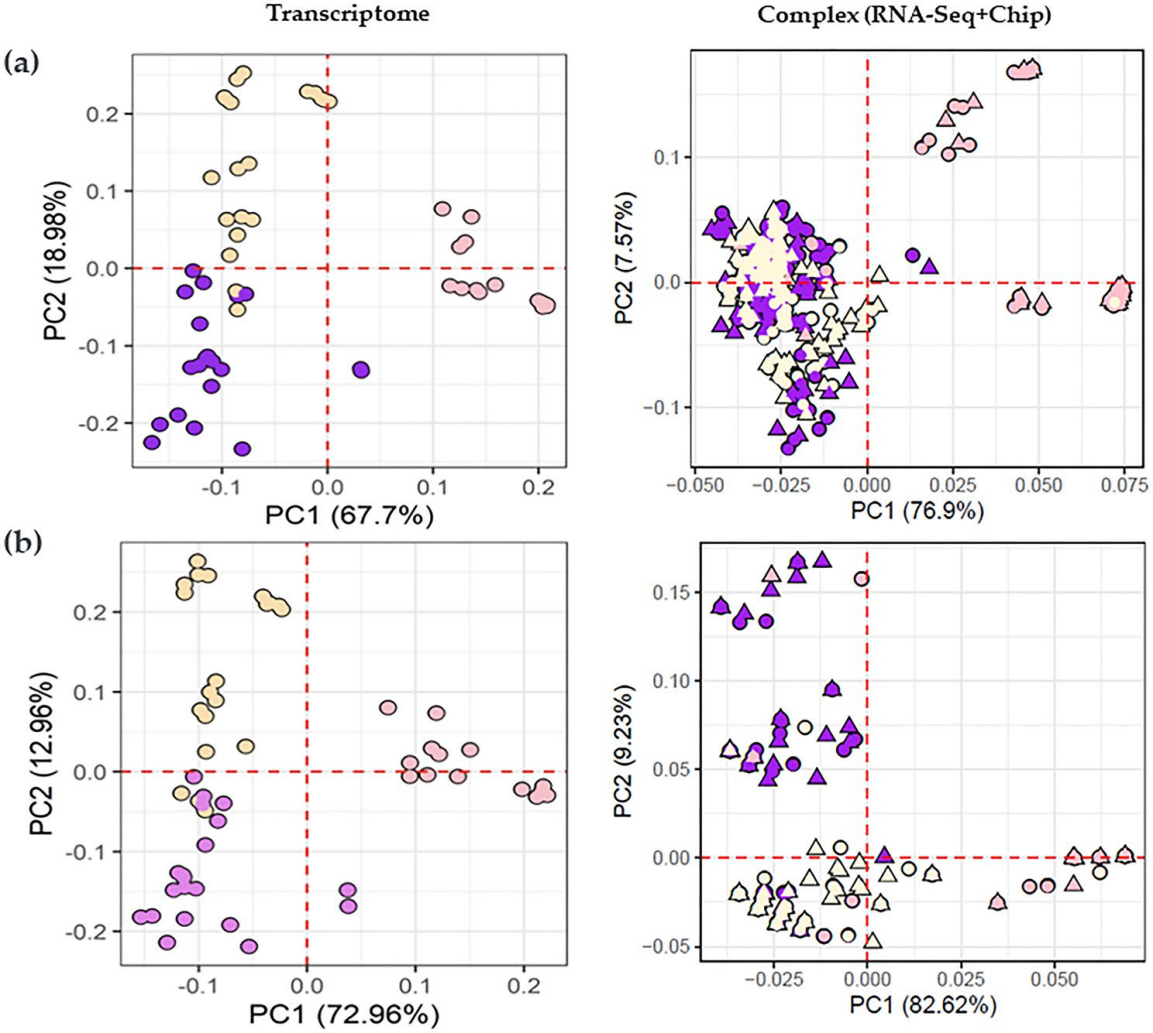
Clustering of SNPs associated with *P. grandiflorus* flower colors. (**a**) Principal compound analysis (PCA) showing the 96 SNPs that represent the flower color classification on the transcriptome and complex dataset. (**b**) PCA showing the 9 SNPs that represent flower color classification using the revised dataset. The colored shapes represent the pink flower (pink), violet flower (violet), white flower (yellow), circle (RNA-Seq), and triangle (Fluidigm chip). The variance percentage of principal components is described in the axis.

PCA analysis showed that the 9 SNPs could be classified into three clusters (i.e., pink, violet, and white) better than the 96 SNPs, even though two SNP groups were well-classified for flower color based on the transcriptome and chip data (Fig. 3). Evaluation of prediction accuracy, which was performed using three criteria, demonstrated that the 9 SNP values was improved than the 96 SNPs (Supplementary Fig. S10). Evaluation of principal components showed low clustering accuracy for color classification in the different SNP subsets (Supplementary Fig. S11). The RF model exhibited higher classification performance than the other models (Table 1 and Fig. 2). Of the different flower color types, pink scored highest in prediction accuracy across the three evaluation factors (i.e., accuracy, sensitivity, and specificity) in both datasets (Supplementary Table S1).

### SNP marker identification and verification

The identified SNP markers were subjected to ML modeling to predict *P. grandiflorus’s* flower color. The 9 selected SNPs correlated strongly with the flower color in *P. grandifloras* and were associated with some secondary metabolite genes, including four pigmentation-related genes, chorismate synthase, and three MYB-like family genes (Table 2).

**Table 2:** Annotation of 9 candidate SNP markers to classify the flower color in *Platycodon grandiflorus.*

ID	Locus*	Region	Variant type	Variant effect	Substitution**	Description
T1	144:153,991	PGJG021990	Missense	Moderate	1127, G>A	Uncharacterized protein
T2	180:177,095	PGJG026810	Synonymous	Low	1101, T>A	Cell division control protein
T3	1316:87,539	PGJG150790	Upstream	Modifier	211, T>A	Myb-like family
T4	4328:246,345	PGJG297010	Downstream	Modifier	82, G>A	Chorismate synthase 1
T5	46:86,514	PGJG007890	Downstream	Modifier	3107, T>A	Uncharacterized protein
T6	4733:14,225	PGJG376590	Synonymous	Low	1989, G>A	Trafficking protein
T7	765:76,395	PGJG097920	Synonymous	Low	852, A>G	Myb-like family
T8	765:78,413	PGJG097920	Missense	Moderate	95, A>T	Myb-like family
T9	1078:54,988	PGJG129730	Synonymous	Low	1539, C>T	Glycosyl hydrolase family

*Scaffold No.: SNP location. **CDS position, allele substitution.

To verify sequence polymorphisms in the predicted region of the 9 SNP candidates, we performed genomic DNA PCR with Sanger sequencing for samples from the three flower colors of *P. grandiflorus.* Our data demonstrate that the 9 SNP candidates can be used as markers to identify flower color in bellflower plants (Table 3). Among the 9 SNP markers, T3 and T7 are double nucleotides in the white flower color accession. For this example, sequence polymorphism in the T3 SNP marker for the three flower colors was distinguished as T (pink), W (white), and A (violet).

**Table 3:** Validation of candidate SNPs by genomic DNA PCR amplification and sequencing.

ID	Primer information		PCR size (bp)	Sequence polymorphism		
	Forward	Reverse		Pink	White	Purple
T1	TGTGCTATCACACCATGTCTTCA	TTAGGGGTCAATCCTACGGTACT	496	T	C	C
T2	GGTGCATCAGAAGAGAACATTCG	GCTAAGTCAGCTCCAACAAATCC	449	A	T	T
T3	AGGTGGAGGTTTTACAATGGCA	CCCAACTCCAGCTTCTTTCCTA	410	T	W(T/A)	A
T4	AGGGATTTATGCATCCAGCAGAT	TTCTTTCTTGTAATGCCCGCTTC	465	T	C	C
T5	TATACATTTGCTGTGGCACCTCT	CCTCTCTCTCCACAACTCTGAAC	398	A	T	T
T6	TCATGCATTTCAGTTTGCATGGT	AGTTTCTTGTGCTGTCCATCAAC	350	T	C	C
T7	TCTCATCACCTTCAGCAGAATCC	GGAGGGAGTAATTAACGAGCCAA	360	C	Y(C/T)	T
T8	GGGAAGAGTACTCGAATAGCTGG	GGTTCTCAAAATTAGGAGGGGGT	535	A	T	T
T9	GGTCCGATGGCAAATGATACAAG	CCCACCACCCATAAGAACTACAA	438	C	T	C

## Discussion

When breeding medicinal plants, breeders perform specific target trait-based selection to improve efficiency. However, traits related to flowering are highly dependent on the plant life cycle. A transcriptome-based SNP approach can efficiently evaluate a specific target trait, such as seed germination, at an early stage^10^. In this study, we designed an approach for identifying SNP molecular markers that consists of RNA sequencing with variant calling, population stratification, association studies, Fluidigm chip experiments, ML modeling, and SNP marker verification (Fig. 1).

ML algorithms are widely used in molecular biology to systematically elucidate specific molecular markers with associated functions and phenotype data^32,33^. Instead of selecting a random classifier for the prediction model development, it is highly recommended to explore multiple classifiers on the same dataset to identify the best classifier^10,32,34,35^. In this regard, we explored support vector machine (SVM), k-neural network (k-NN), random forest (RF), C5.0 decision tree (C5.0), partial least square (PLS), and gradient boosting (GBM). The result shows that the RF-based model achieved the best performance among the six classifiers employed, indicating that RF can capture the hidden relationship between positive and negative samples efficiently compared to other classifiers. When 96 SNPs were validated using dataset 1, the validation efficiency of flower color identification was low, while training accuracy was high. This overfitting problem was improved by the new dataset using the feature importance value of the ML algorithm^36^. Therefore, 9 SNPs were newly identified that improved the overfitting problem and validated using dataset 2.

A transcriptome-based SNP approach can be a cost/time-saving method for identifying large-scale markers^37^. We found 9 transcriptome-wide SNPs in the coding regions associated with flower color (Table 2). Flower color constitutes one phenotype that could be used to identify secondary metabolites such as indole alkaloids (yellow), anthocyanin (blue, violet, and red), and carotenoids (yellow, orange, and red)^38,39^. Among related genes from the selected 9 SNPs, the chorismate synthase gene (i.e., T4 SNP marker) is actively involved in the biosynthesis of anthocyanin, a precursor for various secondary metabolites^30,40^, and is characterized by turmeric leaves variegation^41^. A glycosyl hydrolase family gene (i.e., T9 SNP marker) activates carbohydrate changes in the secondary metabolite gene^42^. Three MYB-like family genes (i.e., T3, T7, and T8 SNP markers) have been reported previously to be involved in pigmentation metabolism in carrot^43^, cherry^44^, and soybean^45^.

SNP markers are the method of choice for plant and animal genetic analyses^46^. Some common SNP genotyping methods used in genetic studies are AS-PCR (allele-specific PCR), CAPS (cleaved amplified polymorphic sequence), and dCAPS (derived CAPS)^47^. An approach for identifying SNP markers using PCR fragments and targeted region sequencing has been reported previously to provide highly accurate SNP genotypes^48^. The 9 SNP sequences were used to search for chromosomal gene location and genomic DNA PCR (Table 3). The 9 SNPs were verified by Sanger sequencing of PCR fragments using primers specific for each marker, and these SNPs were found to be located near those detected by genotyping chip (Supplementary Fig. S12). These 9 SNP markers could be effective candidates for assessing carbohydrate changes in secondary metabolite genes associated with flower color. Therefore, identifying SNPs from the transcriptome using the ML model might represent a useful approach for predicting flower color in *P. grandiflorus*. Results from our study could help select the best trait in edible urban gardening when used in combination with advanced breeding systems such as genome selection, even though plant accessions are highly heterogeneous. Finally, this methodology can be applied to different characteristics which have additional phenotype and genotype datasets.

## Supplementary Information

Supplementary Information.

## Data Availability

All raw sequencing data produced in this study have been deposited into the NCBI Sequence Rad Archive (SRA) under the BioProject number PRJNA632346.
