# Supplementary material for: Machine learning, transcriptome, and genotyping chip analyses provide insights into SNP markers identifying flower color in Platycodon grandiflorus
Source: Sci Rep. 2021 Apr 13;11:8019. doi: 10.1038/s41598-021-87281-0 (PMC8044237; doi:10.1038/s41598-021-87281-0)
Supplement: Supplementary file 1 — Supplementary Information. [file 41598_2021_87281_MOESM1_ESM.docx]

**Supplementary Information**

**Machine learning, transcriptome, and genotyping chip analyses provide insights into SNP markers identifying flower color in *Platycodon grandiflorus***

Go-Eun Yu^1†^, Younhee Shin^2†^, Sathiyamoorthy Subramaniyam^2^, Sang-Ho Kang^1^, Si-Myung Lee^1^, Chuloh Cho^3^, Seung-Sik Lee^4,5^, Chang-Kug Kim^1*^

^1^Genomics Division, National Institute of Agricultural Sciences, Jeonju 54874, Korea

^2^Research and Development Center, Insilicogen Inc., Yongin-si 16954, Gyeonggi-do, Republic of Korea;

^3^Crop Foundation Research Division, National Institute of Crop Science, RDA, Wanju 55365, Korea

^4^Advanced Radiation Technology Institute, Korea Atomic Energy Research Institute, 29 Geumgu-gil, Jeongeup, 56212, Korea

^5^Department of Radiation Science and Technology, University of Science and Technology, Daejeon 34113, Korea


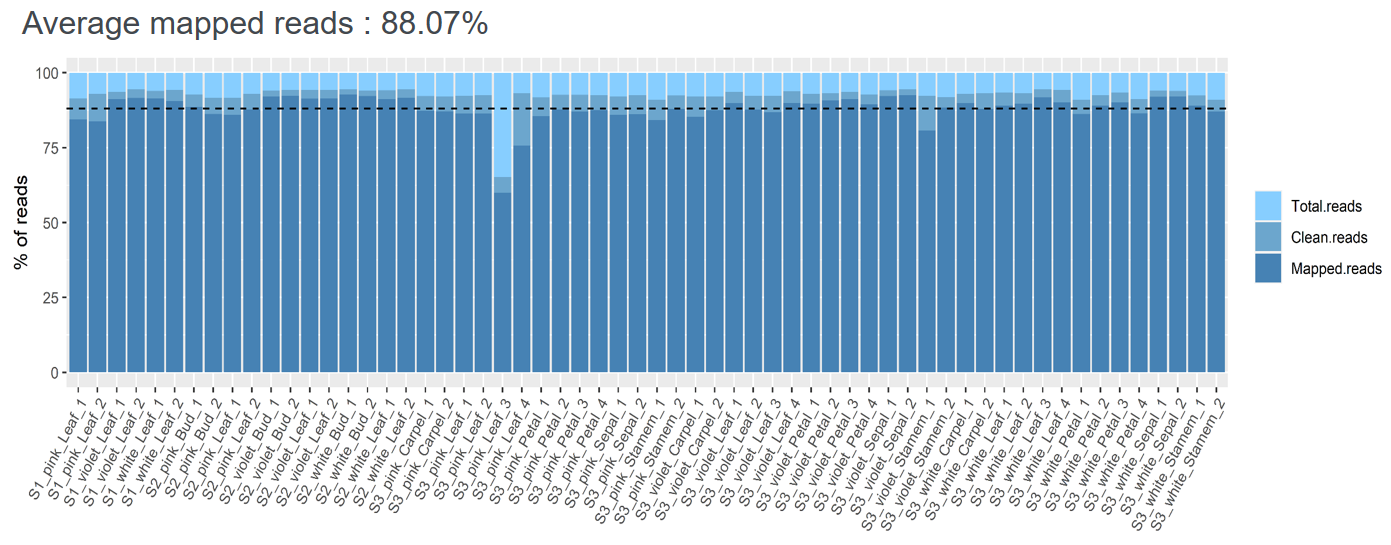


**Supplementary Figure S1.** Mapping ratio of *P. grandiflorus* sample to the reference genome

**
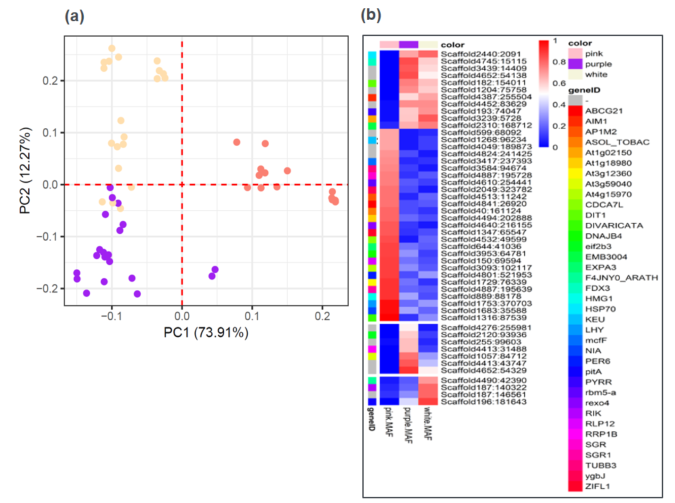
**

**Supplementary Figure S2.** The systematically selected 49 SNPs with TPM ≥ 0.3, Read count ≥ 5, log2FC ≥ 2.0, and 5 KB up/down Flanks. (a) PCA plot. The circle colors represent the pink flower (pink), violet flower (violet), and white flower (yellow). (b) Heat-map for minor allele frequency (MAF). The Heat-Map was created with pheatmap version 1.0.12 with MAF values.

**
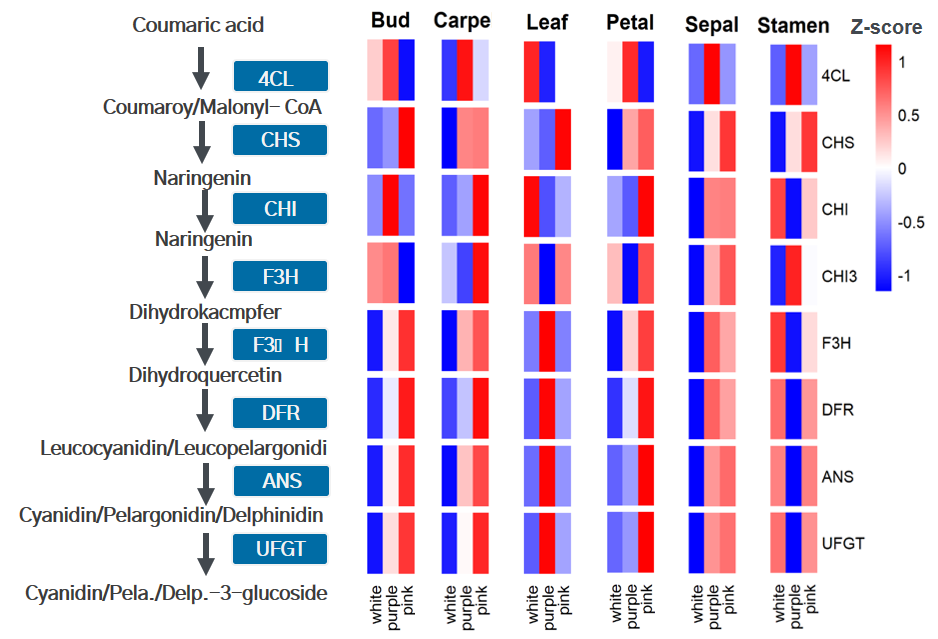
**

**Supplementary Figure S3.** The gene expression profile of the anthocyanin biosynthesis pathway using three flower color accessions. The Heat-Map was created with pheatmap version 1.0.12 with transcripts per million (TPM) values.

**
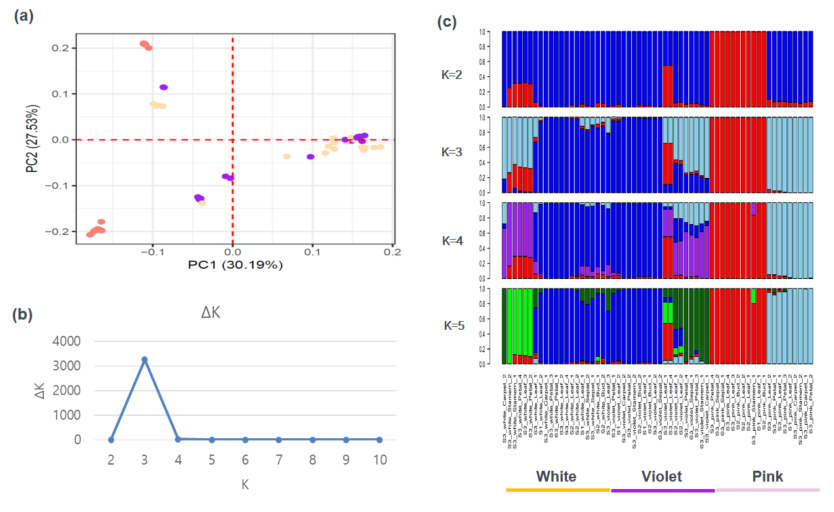
**

**Supplementary Figure S4.** Genetic structures of 60 individual *P. grandiflorus*. (a) Plots of the principle components using 76,629 high-quality SNPs. Individual SNPs are shown as colored circles that represent pink (pink), violet (violet), and white (yellow) populations. The percentage of variance is described in the axis. (b) Delta K (∆K) graph obtained structure analysis at an optimal value of K = 3. (c) Population structure histogram inferred using the Bayesian model-based STRUCTURE clustering method. Each bar represents a specific cluster of three flower color-related populations.

**
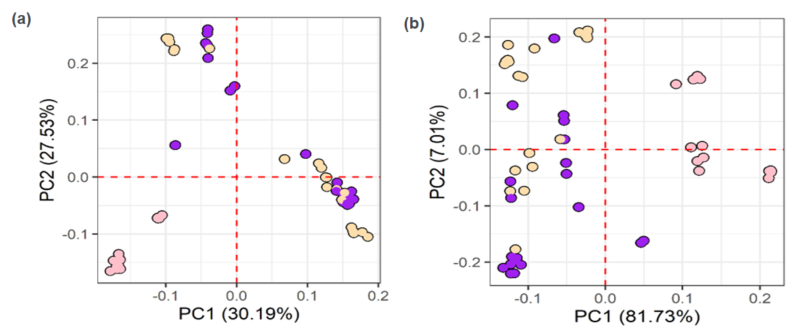
**

**Supplementary Figure S5.** Clustering of SNPs associated with *P. grandiflorus* flower colors. (a) Principle compound analysis (PCA) showing the 76,629 SNPs that represent the three flower colors. (b) PCA showing the 1,224 SNPs that represent flower color. The colored shapes represent the pink flower (pink), violet flower (violet), and white flower (yellow). The SNPs of similar position are omitted and variance percentage of principle components is described in the axis.

**
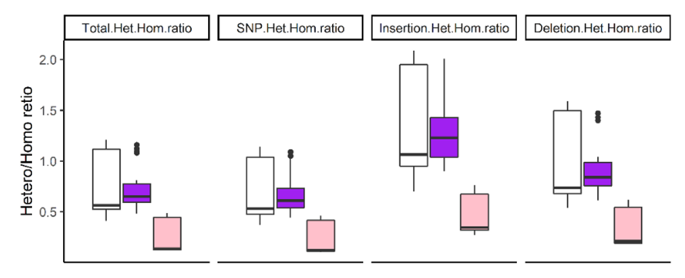
**

**Supplementary Figure S6.** The variation of hetero/homo allele ratio of each flower color group. The colored shapes represent the pink flower (pink), violet flower (violet), and white flower (white). The ratio is estimated by total 1,624,281 variants which consist of 1,391,964 SNPs, 110,687 insertion polymorphisms, and 121,630 deletion polymorphisms.

**
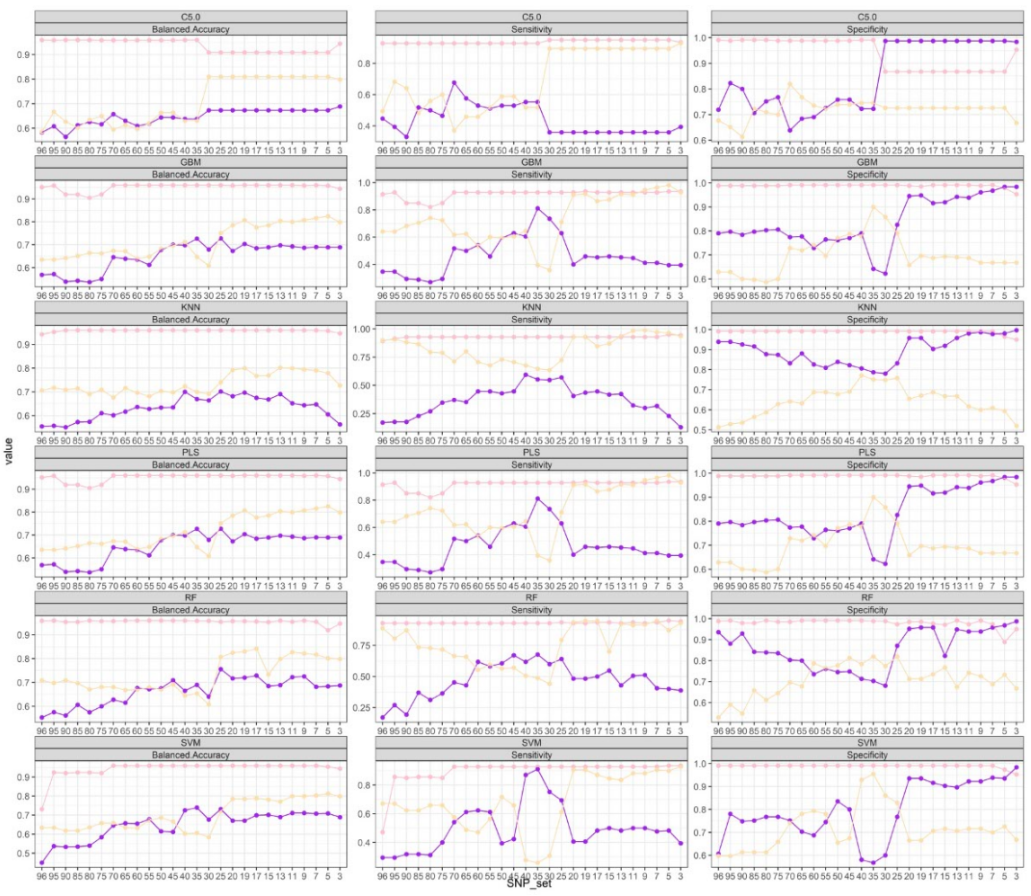
**

**Supplementary Figure S7.** The varying pattern of prediction accuracies of the six machine learning models along with sensitivity and specificity factor values, while using different SNP subsets.

**
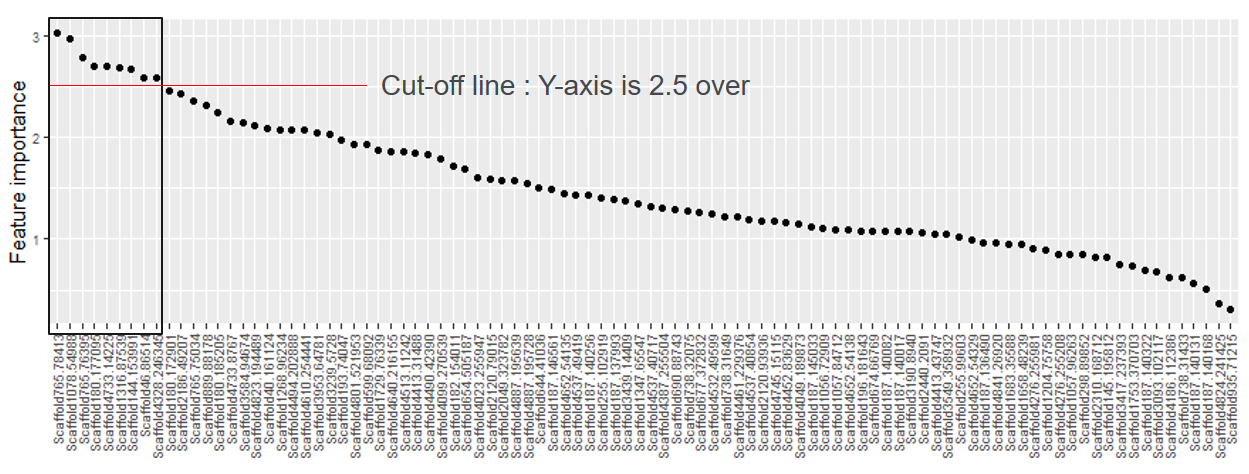
**

**Supplementary Figure S8.** The feature importance values predicted using the random forest model.

**
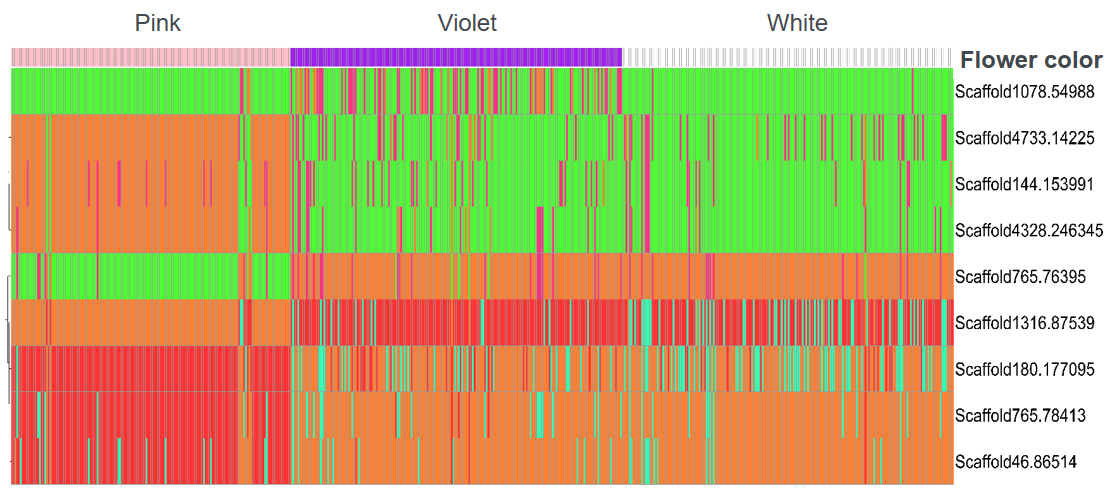
**

**Supplementary Figure S9.** Positional bi-allele heat-map representation of selected 9 SNPs from all the genotyped accessions in *P. grandiflorus*. The Heat-Map was created with pheatmap version 1.0.12.

**
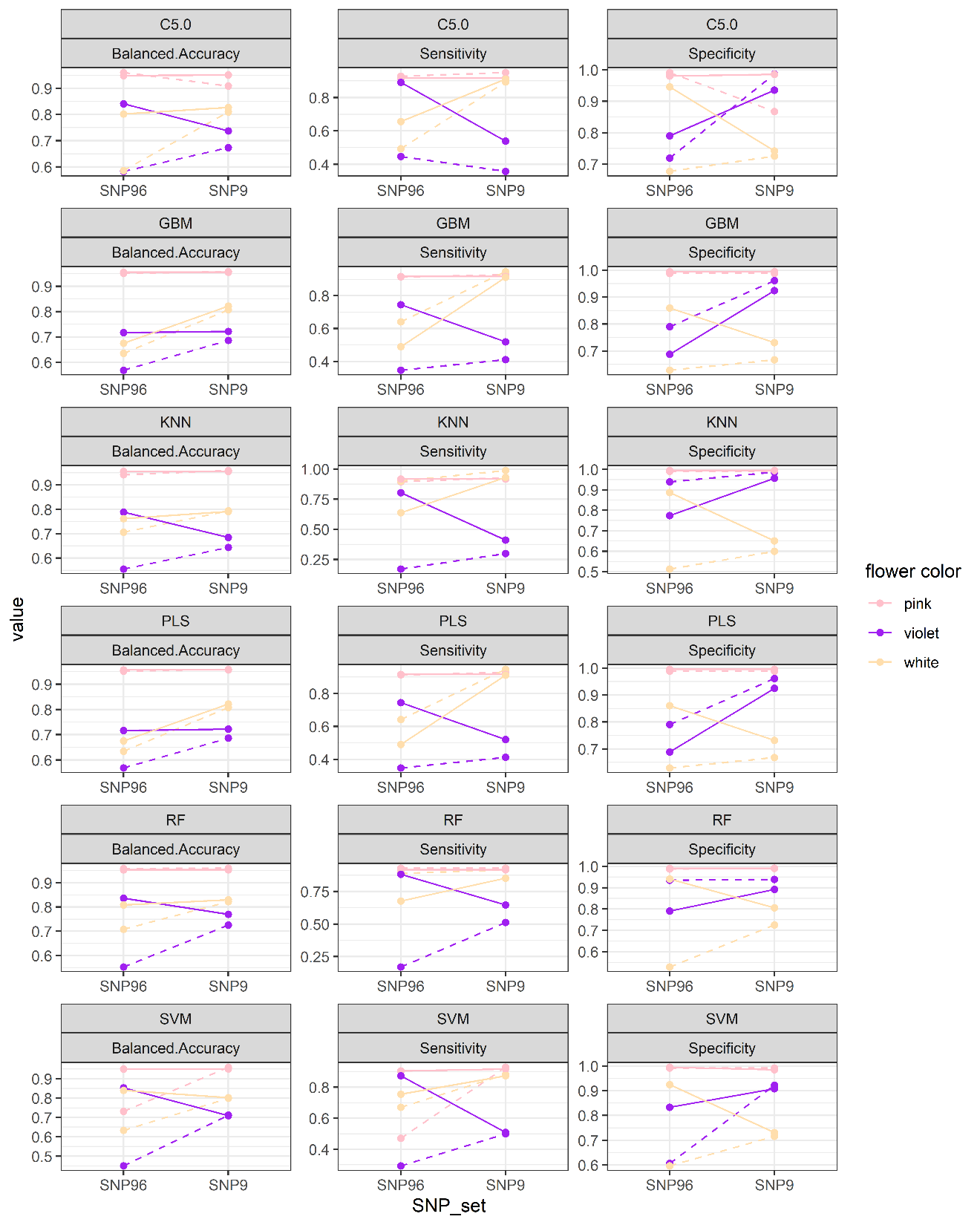
**

**Supplementary Figure S10.** The varying pattern of prediction accuracy of the six machine learning models along with sensitivity and specificity factor values using the 96 SNPs and 9 SNPs. The dotted line represents dataset 1 and the solid line represents dataset 2.

**
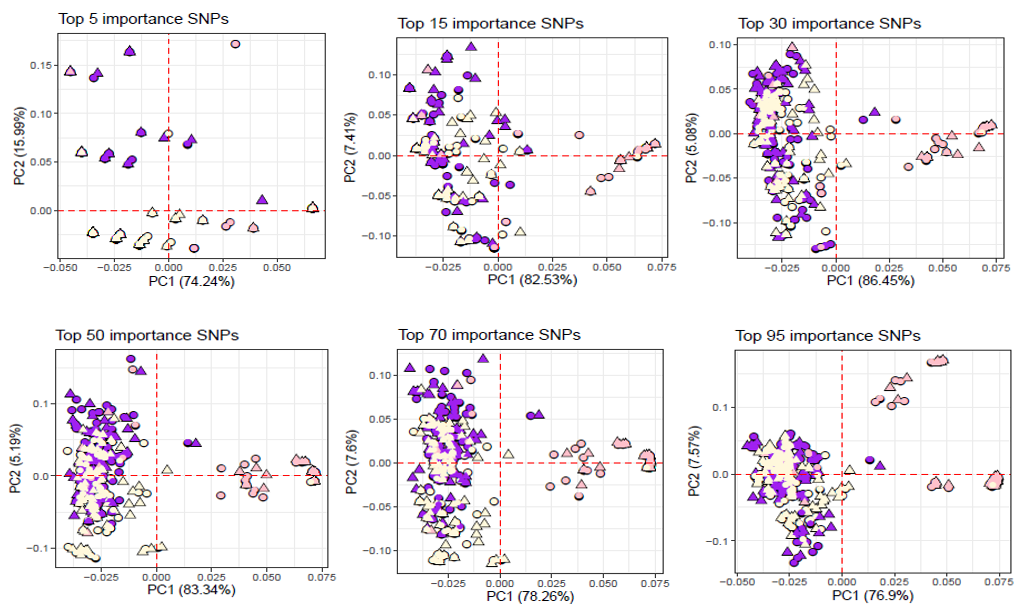
**

**Supplementary Figure S11.** Clustering of SNPs associated with *P. grandiflorus* flower colors. Principle compound analysis (PCA) showing the 5, 15, 30, 50, 70, and 95 SNPs that represent the flower color classification on the complex dataset 2. The colored shapes represent the pink flower (pink), violet flower (violet), white flower (yellow), circle (RNA-Seq), and triangle (Fluidigm chip). The variance percentage of principle components is described in the axis.


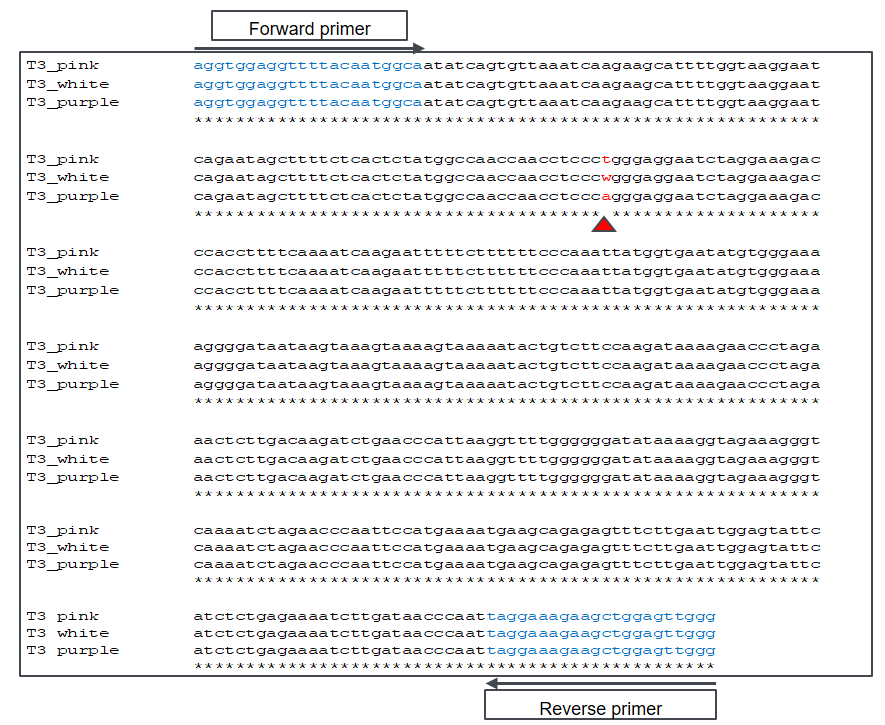


**Supplementary Figure S12.** Sequence comparison of PCR products amplified using primer pair T3 SNP. Genomic DNA PCRs were performed using genomic DNA templates extracted from plants with flowers of pink, white, and violet (purple) color and the amplified PCR products were directly sequenced by the Sanger sequencing method using the ABI 3730xl System. Candidate SNP and the used primers are indicated by a red triangle and arrows, respectively.

**Supplementary Table 1.** Predictions accuracy of six ML models on the two datasets.

| Classification | | | First (96 Features SNPs) | | | Second (9 Features SNPs) | | |
| --- | --- | --- | --- | --- | --- | --- | --- | --- |
| Type | Color | Model | Accuracy | Sensitivity | Specificity | Accuracy | Sensitivity | Specificity |
| Dataset1 | Pink | GBM | 0.951 | 0.914 | 0.988 | 0.958 | 0.929 | 0.988 |
|  |  | SVM | 0.731 | 0.471 | 0.991 | 0.960 | 0.929 | 0.991 |
|  |  | RF | 0.958 | 0.929 | 0.988 | 0.960 | 0.929 | 0.991 |
|  |  | KNN | 0.942 | 0.893 | 0.991 | 0.960 | 0.929 | 0.991 |
|  |  | C5.0 | 0.960 | 0.929 | 0.991 | 0.909 | 0.950 | 0.868 |
|  |  | PLS | 0.951 | 0.914 | 0.988 | 0.958 | 0.929 | 0.988 |
|  | Violet | GBM | 0.569 | 0.347 | 0.790 | 0.687 | 0.412 | 0.961 |
|  |  | SVM | 0.450 | 0.294 | 0.606 | 0.711 | 0.500 | 0.923 |
|  |  | RF | 0.553 | 0.171 | 0.935 | 0.725 | 0.512 | 0.939 |
|  |  | KNN | 0.555 | 0.171 | 0.939 | 0.644 | 0.300 | 0.987 |
|  |  | C5.0 | 0.583 | 0.447 | 0.719 | 0.673 | 0.359 | 0.987 |
|  |  | PLS | 0.569 | 0.347 | 0.790 | 0.687 | 0.412 | 0.961 |
|  | White | GBM | 0.635 | 0.641 | 0.629 | 0.807 | 0.947 | 0.668 |
|  |  | SVM | 0.634 | 0.671 | 0.597 | 0.799 | 0.882 | 0.716 |
|  |  | RF | 0.709 | 0.888 | 0.529 | 0.822 | 0.918 | 0.726 |
|  |  | KNN | 0.706 | 0.900 | 0.513 | 0.794 | 0.988 | 0.600 |
|  |  | C5.0 | 0.586 | 0.494 | 0.677 | 0.810 | 0.894 | 0.726 |
|  |  | PLS | 0.635 | 0.641 | 0.629 | 0.807 | 0.947 | 0.668 |
| Dataset2 | Pink | GBM | 0.956 | 0.917 | 0.995 | 0.956 | 0.917 | 0.995 |
|  |  | SVM | 0.950 | 0.905 | 0.995 | 0.951 | 0.917 | 0.985 |
|  |  | RF | 0.953 | 0.917 | 0.990 | 0.953 | 0.917 | 0.990 |
|  |  | KNN | 0.956 | 0.917 | 0.995 | 0.956 | 0.917 | 0.995 |
|  |  | C5.0 | 0.949 | 0.917 | 0.980 | 0.951 | 0.917 | 0.985 |
|  |  | PLS | 0.956 | 0.917 | 0.995 | 0.956 | 0.917 | 0.995 |
|  | Violet | GBM | 0.717 | 0.745 | 0.688 | 0.722 | 0.520 | 0.925 |
|  |  | SVM | 0.853 | 0.873 | 0.833 | 0.709 | 0.510 | 0.909 |
|  |  | RF | 0.836 | 0.882 | 0.790 | 0.770 | 0.647 | 0.892 |
|  |  | KNN | 0.789 | 0.804 | 0.774 | 0.684 | 0.412 | 0.957 |
|  |  | C5.0 | 0.841 | 0.892 | 0.790 | 0.737 | 0.539 | 0.935 |
|  |  | PLS | 0.717 | 0.745 | 0.688 | 0.722 | 0.520 | 0.925 |
|  | White | GBM | 0.675 | 0.490 | 0.860 | 0.821 | 0.912 | 0.731 |
|  |  | SVM | 0.840 | 0.755 | 0.925 | 0.802 | 0.873 | 0.731 |
|  |  | RF | 0.809 | 0.676 | 0.941 | 0.830 | 0.853 | 0.806 |
|  |  | KNN | 0.762 | 0.637 | 0.887 | 0.791 | 0.931 | 0.651 |
|  |  | C5.0 | 0.802 | 0.657 | 0.946 | 0.827 | 0.912 | 0.742 |
|  |  | PLS | 0.675 | 0.490 | 0.860 | 0.821 | 0.912 | 0.731 |

**Supplementary Table 2.** Overview of 60 samples from each tissue on the flower color types

| Type | Tissue | Flowering  - 7 Day | Flowering | Flowering  + 7 Day | Number |
| --- | --- | --- | --- | --- | --- |
| Pink | Bud | 0 | 2 | 0 | 20 |
|  | Carpel | 0 | 0 | 2 |  |
|  | Leaf | 2 | 2 | 4 |  |
|  | Petal | 0 | 0 | 4 |  |
|  | Sepal | 0 | 0 | 2 |  |
|  | Stamen | 0 | 0 | 2 |  |
| Violet | Bud | 0 | 2 | 0 | 20 |
|  | Carpel | 0 | 0 | 2 |  |
|  | Leaf | 2 | 2 | 4 |  |
|  | Petal | 0 | 0 | 4 |  |
|  | Sepal | 0 | 0 | 2 |  |
|  | Stamen | 0 | 0 | 2 |  |
| White | Bud | 0 | 2 | 0 | 20 |
|  | Carpel | 0 | 0 | 2 |  |
|  | Leaf | 2 | 2 | 4 |  |
|  | Petal | 0 | 0 | 4 |  |
|  | Sepal | 0 | 0 | 2 |  |
|  | Stamen | 0 | 0 | 2 |  |

**Supplementary Table 3.** Overview of samples and machine learning (ML) datasets.

| **Color type** | **RNA-Seq** | **Fluidigm Chip** |
| --- | --- | --- |
| **Pink (P)** | **20** | **140** |
| **Violet (V)** | **20** | **170** |
| **White (W)** | **20** | **170** |
| **Total** | **60** | **480** |
| **ML Data Set** | **Training** | **Validation** |
| **Dataset1** | **60** | **480** |
| **Dataset2** | **252*** | **288**** |

*RNA-Seq(60, All) + Chip (Pink:56+Violet:68+White:68)

**Chip (Pink:84+Violet:102+White:102)

**Supplementary Table 4.** Annotation of selected 96 SNPs to classify the flower color in Platycodon grandiflorus.

| **SNP ID** | **Region** | **Variant type** | **Substitution** | **Symbol** |
| --- | --- | --- | --- | --- |
| Scaffold1057:84712 | PGJG127620 | synonymous | 615, ,A>G | At3g59040 |
| Scaffold1204:75758 | PGJG141820 | upstream | 178, T>A | - |
| Scaffold1268:96234 | PGJG146990 | downstream | 188, T>A | KEU |
| Scaffold1316:87539 | PGJG150790 | upstream | 211, T>A | DIVARICATA |
| Scaffold1347:65547 | PGJG152740 | downstream | 71, C>T | ZIFL1 |
| Scaffold1683:35588 | PGJG176500 | upstream | 225, C>G | NIA |
| Scaffold1729:76339 | PGJG179940 | downstream | 2489, A>G | At3g12360 |
| Scaffold1753:370703 | PGJG181800 | upstream | 267, G>C | LHY |
| Scaffold182:154011 | PGJG027600 | downstream | 9, C>A | DIT1 |
| Scaffold187:140322 | PGJG028390 | intron | 1623, C>T | RIK |
| Scaffold187:146561 | PGJG028400 | upstream | 3507, A>T | - |
| Scaffold193:74047 | PGJG028990 | upstream | 3210, T>G | PYRR |
| Scaffold196:181643 | PGJG029500 | downstream | 20, A>G | pitA |
| Scaffold2049:323782 | PGJG200650 | downstream | 60, C>G | ygbJ |
| Scaffold2120:93936 | PGJG204660 | synonymous | 57, G>C | eif2b3 |
| Scaffold2310:168712 | PGJG216250 | upstream | 75, T>C | EMB3004 |
| Scaffold2440:2091 | PGJG223470 | downstream | 10, G>C | HSP70 |
| Scaffold255:99603 | PGJG036980 | downstream | 1024, C>G | - |
| Scaffold3093:102117 | PGJG251770 | downstream | 2598, T>C | At4g15970 |
| Scaffold3239:5728 | PGJG257290 | upstream | 306, T>C | At1g02150 |
| Scaffold3417:237393 | PGJG263610 | downstream | 3078, C>T | mcfF |
| Scaffold3439:14409 | PGJG264770 | downstream | 3461, T>C | - |
| Scaffold3584:94674 | PGJG269980 | downstream | 267, A>T | TUBB3 |
| Scaffold3953:64781 | PGJG279130 | downstream | 338, C>T | DNAJB4 |
| Scaffold40:161124 | PGJG007080 | downstream | 2195, C>T | ASOL_TOBAC |
| Scaffold4049:189873 | PGJG283430 | downstream | 1599, A>G | - |
| Scaffold4276:255981 | PGJG294190 | missense | 397, G>A | - |
| Scaffold4387:255504 | PGJG308150 | downstream | 129, C>T | AIM1 |
| Scaffold4413:31488 | PGJG313420 | missense | 1462, G>A | RRP1B |
| Scaffold4413:43747 | PGJG313430 | synonymous | 324, G>A | - |
| Scaffold4452:83629 | PGJG321830 | downstream | 57, T>A | - |
| Scaffold4490:42390 | PGJG331210 | downstream | 91, C>G | F4JNY0_ARATH |
| Scaffold4494:202888 | PGJG331850 | downstream | 1392, T>C | At1g18980 |
| Scaffold4513:11242 | PGJG335350 | upstream | 8, C>T | AP1M2 |
| Scaffold4532:49599 | PGJG339160 | downstream | 679, A>G | CDCA7L |
| Scaffold4610:254441 | PGJG354080 | upstream | 1217, A>C | rbm5-a |
| Scaffold4640:216155 | PGJG359280 | downstream | 1412, T>C | rexo4 |
| Scaffold4652:54138 | PGJG361580 | upstream | 4929, A>G | - |
| Scaffold4652:54329 | PGJG361600 | upstream | 253, A>G | - |
| Scaffold4745:15115 | PGJG379330 | upstream | 3361, A>G | FDX3 |
| Scaffold4801:521953 | PGJG388520 | downstream | 1051, A>C | PER6 |
| Scaffold4824:241425 | PGJG391940 | upstream | 143, G>A | - |
| Scaffold4841:26920 | PGJG394380 | upstream | 131, C>G | ABCG21 |
| Scaffold4887:195639 | PGJG401390 | downstream | 4950, A>G | SGR1 |
| Scaffold4887:195728 | PGJG401400 | downstream | 4020, A>G | SGR |
| Scaffold599:68092 | PGJG080140 | upstream | 48, C>T | - |
| Scaffold644:41036 | PGJG084680 | downstream | 3497, G>T | EXPA3 |
| Scaffold889:88178 | PGJG111050 | downstream | 13, A>T | HMG1 |
| Scaffold1057:96263 | PGJG127620 | synonymous | 1656, T>C | At3g59040 |
| Scaffold144:153991 | PGJG021990 | missense | 1127, G>A | - |
| Scaffold145:195812 | PGJG022140 | upstream | 569, A>G | - |
| Scaffold180:172901 | PGJG026810 | upstream | 1592, C>A | CDC48C |
| Scaffold180:177095 | PGJG026810 | synonymous | 1101, T>A | CDC48C |
| Scaffold180:185205 | PGJG026810 | synonymous | 2295, T>C | CDC48C |
| Scaffold187:136490 | PGJG028390 | intron | 949+110, G>A | RIK |
| Scaffold187:137993 | PGJG028390 | intron | 1623+360, A>G | RIK |
| Scaffold187:140017 | PGJG028390 | intron | 1623+2384, T>C | RIK |
| Scaffold187:140082 | PGJG028390 | intron | 1623+2449, T>G | RIK |
| Scaffold187:140131 | PGJG028390 | intron | 1623+2498, A>G | RIK |
| Scaffold187:140168 | PGJG028390 | intron | 1623+2535, C>T | RIK |
| Scaffold187:140256 | PGJG028390 | intron | 1623+2623, T>C | RIK |
| Scaffold187:145033 | PGJG028390 | intron | 1624-4069, T>C | RIK |
| Scaffold2186:49207 | PGJG208050 | downstream | 182, C>T | - |
| Scaffold4023:255947 | PGJG281850 | downstream | 2621, C>T | STT3B |
| Scaffold4186:112386 | PGJG288930 | upstream | 3179, A>G | Q8RWU7_ARATH |
| Scaffold4328:246345 | PGJG297010 | downstream | 82, G>A | CS1 |
| Scaffold46:86514 | PGJG007890 | downstream | 3107, T>A | - |
| Scaffold4652:54135 | PGJG361580 | upstream | 4926, A>T | - |
| Scaffold4733:14225 | PGJG376590 | synonymous | 1989, G>A | TRAPPC8 |
| Scaffold4733:8767 | PGJG376590 | missense | 3158, C>G | TRAPPC8 |
| Scaffold4823:194489 | PGJG391740 | splice | 71+7, T>C | - |
| Scaffold654:505187 | PGJG085570 | synonymous | 897, C>T | At1g50460 |
| Scaffold765:75034 | PGJG097920 | synonymous | 1431, T>C | SWI3B |
| Scaffold765:76395 | PGJG097920 | synonymous | 852, A>G | SWI3B |
| Scaffold765:78413 | PGJG097920 | missense | 95, A>T | SWI3B |
| Scaffold935:71215 | PGJG116710 | downstream | 442, T>A | - |
| Scaffold255:102919 | PGJG036980 | intron | 440-1666, A>T | - |
| Scaffold298:89852 | PGJG042430 | missense | 199, G>C | - |
| Scaffold674:66769 | PGJG087480 | downstream | 17, T>G | - |
| Scaffold677:372832 | PGJG088230 | synonymous | 426, C>T | RECQL3 |
| Scaffold690:88743 | PGJG089430 | synonymous | 408, A>G | PMR5 |
| Scaffold738:31433 | PGJG095210 | downstream | 81, C>A | RPS3C |
| Scaffold738:31649 | PGJG095210 | synonymous | 165, T>C | RPS3C |
| Scaffold738:32075 | PGJG095210 | upstream | 145, G>A | RPS3C |
| Scaffold1056:72909 | PGJG127480 | downstream | 4732, T>A | - |
| Scaffold1078:54988 | PGJG129730 | synonymous | 1539, C>T | BXL6 |
| Scaffold1658:48239 | PGJG174910 | synonymous | 480, C>T | - |
| Scaffold2120:79015 | PGJG204660 | downstream | 78, A>G | eif2b3 |
| Scaffold3190:8940 | PGJG255590 | synonymous | 348, A>G | At1g10890 |
| Scaffold3549:358932 | PGJG267740 | synonymous | 177, C>G | FTR-A |
| Scaffold4099:270539 | PGJG285130 | synonymous | 435, T>C | GST23_MAIZE |
| Scaffold4276:255208 | PGJG294170 | upstream | 4212, C>T | - |
| Scaffold4461:229376 | PGJG324060 | synonymous | 1164, G>A | - |
| Scaffold4537:40717 | PGJG339890 | synonymous | 120, A>G | LKR/SDH |
| Scaffold4537:40854 | PGJG339890 | missense | 257, C>G | LKR/SDH |
| Scaffold4537:49419 | PGJG339890 | synonymous | 1686, C>T | LKR/SDH |

*Scaffold No:SNP location. **CDS position, allele substitution
